# Miro1 deficiency in amyotrophic lateral sclerosis

**DOI:** 10.3389/fnagi.2015.00100

**Published:** 2015-05-26

**Authors:** Fan Zhang, Wenzhang Wang, Sandra L. Siedlak, Yingchao Liu, Jun Liu, Keji Jiang, George Perry, Xiongwei Zhu, Xinglong Wang

**Affiliations:** ^1^Department of Pathology, Case Western Reserve UniversityCleveland, OH, USA; ^2^Department of Neurosurgery, Shandong Provincial Hospital, Shandong UniversityJinan, China; ^3^Department of Neurology and Institute of Neurology, Ruijin Hospital, Shanghai Jiao Tong University School of MedicineShanghai, China; ^4^East China Sea Fisheries Research Institute, Chinese Academy of Fishery SciencesShanghai, China; ^5^College of Sciences, University of Texas at San AntonioSan Antonio, TX, USA

**Keywords:** amyotrophic lateral sclerosis, Miro1, mitochondrial transport, SOD1, TDP-43, glutamate excitotoxicity, neurodegeneration

## Abstract

Proper transportation of mitochondria to sites with high energy demands is critical for neuronal function and survival. Impaired mitochondrial movement has been repeatedly reported in motor neurons of amyotrophic lateral sclerosis (ALS) patients and indicated as an important mechanism contributing to motor neuron degeneration in ALS. Miro1, a RhoGTPase also referred to as Rhot1, is a key regulator of mitochondrial movement linking mitochondria and motor proteins. In this study, we investigated whether the expression of Miro1 was altered in ALS patients and ALS animal models. Immunoblot analysis revealed that Miro1 was significantly reduced in the spinal cord tissue of ALS patients. Consistently, the decreased expression of Miro1 was also noted only in the spinal cord, and not in the brain tissue of transgenic mice expressing ALS-associated SOD1 G93A or TDP-43 M337V. Glutamate excitotoxicity is one of the major pathophysiological mechanisms implicated in the pathogenesis of ALS, and we found that excessive glutamate challenge lead to significant reduction of Miro1 expression in spinal cord motor neurons both *in vitro* and in mice. Taken together, these findings show Miro1 deficiency in ALS patients and ALS animal models and suggest glutamate excitotoxicity as a likely cause of Miro1 deficiency.

## Introduction

Amyotrophic lateral sclerosis (ALS) is one of the most common motor neuron diseases characterized by progressive neurodegeneration of motor neurons in the brainstem and spinal cord (Rowland and Shneider, 2001). The vast majority of ALS cases, referred to as sporadic ALS (sALS), are not genetically transmitted and their causes remain enigmatic. 5–10% of ALS cases are familial (fALS), of which most are associated with repeat expansions of the C9ORF72 gene or mutations in genes encoding copper–zinc superoxide dismutase (SOD1), TAR DNA binding protein 43 (TDP-43) or fused in sarcoma (FUS; Al-Chalabi et al., 2012). The cellular and molecular mechanisms underlying motor neuron loss in both fALS and sALS are unknown, and effective treatments are extremely limited.

Unlike any other cell type, neurons especially motor neurons with extended axons and dendrites, are highly dependent on mitochondrial trafficking for survival, because synaptic transmission and ion channel activity in distal synaptic terminals are all great energy taxing processes (Kann and Kovács, 2007), and mitochondria need to be efficiently transported to these sites with high metabolic requirement (Sheng, 2014). Interestingly, accumulation of mitochondria was reported in the proximal axons of motor neurons of ALS patients, indicating the possible impairment of mitochondrial transport (Sasaki and Iwata, 1996). Consistently, impaired mitochondrial movement and altered mitochondrial distribution were noted in motor neurons expressing ALS-associated mutant SOD1 G93A or TDP-43 M337V *in vitro* and in mice (De Vos et al., 2007; Bilsland et al., 2010; Wang et al., 2013; Magrané et al., 2014). Despite expanding evidence for the important role of mitochondrial trafficking deficits in the pathogenesis of ALS (Shi et al., 2010), the underlying mechanism is still not clear.

Mitochondria are transported bidirectionally in neurites along microtubules for fast movement and along actin filaments for slow movement via different motor-adaptor complexes. In axons with uniform plus-end-out microtubules, mitochondrial anterograde transport is driven by kinesin motors whereas retrograde movement is controlled by dynein motors (Schwarz, 2013). Microtubules in dendrites have mixed orientation, therefore mitochondria can move in either the anterograde or the retrograde direction (Sheng and Cai, 2012). Motor proteins are usually indirectly linked to mitochondria by Miro1-Milton adaptor complex, in which Miro1 is the only known mitochondrial outer membrane protein directly coupling mitochondria and motor-adaptor complexes (Schwarz, 2013). Interestingly, a most recent study in mice found that motor neurons are vulnerable to Miro-1 ablation, and this Miro1 deficiency is sufficient to specifically cause motor neuron degeneration and symptoms of motor neuron diseases (Nguyen et al., 2014). Since ALS patients and ALS experimental models display impaired mitochondrial movement, here we sought to investigate whether the expression of Miro-1 is altered in patients and experimental models of ALS, the most common motor neuron disease.

## Materials and Methods

### Embryonic Primary Motor Neuron Isolation and Culture

Timed pregnant Sprague–Dawley rats from Charles River were sacrificed following the protocol approved by the Institutional Animal Care and Use Committee (IACUC) at Case Western Reserve University. E13–15 rat embryos were obtained and primary motor neurons were isolated as described (Wang et al., 2015). Taken briefly, spinal cords were dissected out in L15 medium without phenol red (Invitrogen) and stored in 2% B27 (Invitrogen) supplemented Hibernate E. After the removal of dorsal root ganglia and the meninges, spinal cords were digested in 0.25% trypsin for 15 min at room temperature followed by Optiprep (Sigma) gradient centrifugation. Glia cells were cultured in Neurobasal medium (Invitrogen) supplemented with 2% B27 (Invitrogen) and 1% GlutaMax (Invitrogen) to obtain glia-conditioned media. After seeding with Neurobasal medium (Invitrogen) supplemented with 2% B27 (Invitrogen), 1% GlutaMax (Invitrogen) and 1% penicillin–streptomycin, motor neurons were cultured in glia-conditioned medium supplemented with 2% horse serum (Sigma), and 10 ng/ml each BDNF, CNTF, and GDNF (Peprotech). The culture medium was half-changed every 3 days. Glia cells or motor neurons were cultured at 37°C in a humidified 5% CO_2_ containing atmosphere.

### Western Blot Analysis, Antibodies and Chemicals

Tissues or culture motor neurons were lysed with 1XCell Lysis Buffer (Cell Signaling Technology, Beverly, MA, USA), plus 1 mM phenylmethylsulfonyl fluoride (Sigma) and Protease Inhibitor Cocktail (Sigma). Equal amounts of total protein extract (10 ug) were resolved by sodium dodecyl sulfate Polyacrylamide gel electrophoresis (SDS-PAGE) and transferred to Immobilon-P (Millipore). Following blocking with 10% non-fat dry milk, primary and secondary antibodies were applied and the blots developed with Immobilon Western Chemiluminescent HRP Substrate (Millipore). Frozen human thoracic spinal cord tissues from 6 age-matched normal (age 58 3.8; sex, M:F = 5:1) and 8 patients with sALS (age 57 3.9; sex, M:F = 6:2) were obtained from NICHD (The Eunice Kennedy Shriver National Institute of Child Health and Human Development) Brain and Tissue Bank. B6SJL-Tg (SOD1*G93A) 1Gur/J (SOD1G93A) mice and C57BL/6-Tg (Prnp-TARDBP*M337V) 4Ptrc/J (TDP-43M337V) mice were obtained from the Jackson Laboratory. Primary antibodies used included mouse anti-RHOT1 for detecting Miro1 (Abnova), mouse anti-VDAC1 (Abcam), rabbit anti-TDP-43 (Proteintech), mouse anti-human TDP-43 (Novus), rabbit anti-SOD1 (Santa Cruz), mouse anti-actin (Millipore, Billerica, MA) and rabbit anti-glyceraldehyde-3-phosphate dehydrogenase (GAPDH) (Cell Signaling). Glutamate (Sigma) and MK-801 (Tocris) were also obtained.

### Immunocytochemistry of Human Spinal Cord

Formalin fixed human spinal cord tissues obtained postmortem from University Hospitals of Cleveland and the NICHD Brain and Tissue Bank were paraffin embedded and sectioned and immunocytochemistry was performed by the peroxidase anti-peroxidase protocol as described (Zhu et al., 2000). Taken briefly, following immersion in xylene, hydration through graded ethanol solutions and elimination of endogenous peroxidase activity by incubation in 3% hydrogen peroxide for 30 min, sections were incubated for 30 min at room temperature in 10% normal goat serum (NGS) in Tris-buffered saline (TBS; 50 mM Tris-HCl, 150 mM NaCl, pH 7.6) to reduce non-specific binding. After rinsing with 1% NGS/TBS, the sections were sequentially incubated overnight at 4°C with mouse anti-COX1 (Abcam, Cambridge, MA). The sections were then incubated in goat anti-mouse antisera (ICN), followed by species-specific peroxidase anti-peroxidase complex (Sternberger Monoclonals and ICN). 3-3′-Diaminobenzidine (DAB) was used as a chromagen.

### Animal Surgery and Glutamate Infusion

Mice surgery and procedures approved by the IACUC at Case Western Reserve University were performed according to the NIH guidelines. 24 h before implantation, mini-osmotic pumps (Model 2001, Alzet, Cupertino, CA; flow rate of 1 μl/h) and brain infusion cannula attached with catheter tubes (Brain infusion kit 3, Alzet, Cupertino) were filled with artificial cerebrospinal fluid (aCSF: 124 mM NaCl, 25 mM NaHCO_3_, 10 mM D-glucose, 2.5 mM KCl, 1 mM MgCl_2_, 2 mM CaCl_2_ and 1 mM NaH_2_PO_4_, adjusted to pH 7.2–7.4 using NaOH) or aCSF containing 10 mM glutamate followed by pump incubation in phosphate buffered saline (PBS) at 37°C overnight. Under avertin anesthesia, a small incision was first made in male mice to expose skull and bregma and mini-osmotic pumps connected with catheter were implanted subcutaneously. Then a hole was drilled in the skull (relative to bregma: AP −0.2 mm, ML 1 mm) and the cannula was positioned 2 mm above the lateral ventricle. Another two holes were drilled by the edge of cannula and self-tapping bone screws (MD-1310, BASi, West Lafayette, IN) were screwed followed by cement. Seven day after surgery, mice were deeply anesthetized by avertin followed by spinal cord and brain tissue collection.

### Statistical Analysis

All data are presented as mean ± sem. Data are analyzed by either student-*t*-test or one-way ANOVA followed by Tukey’s multiple comparison test. Statistical difference was considered significant if *p* < 0.05.

## Results

### Reduced Expression of Miro1 in Spinal Cords of sALS Patients

**Figure 1:**
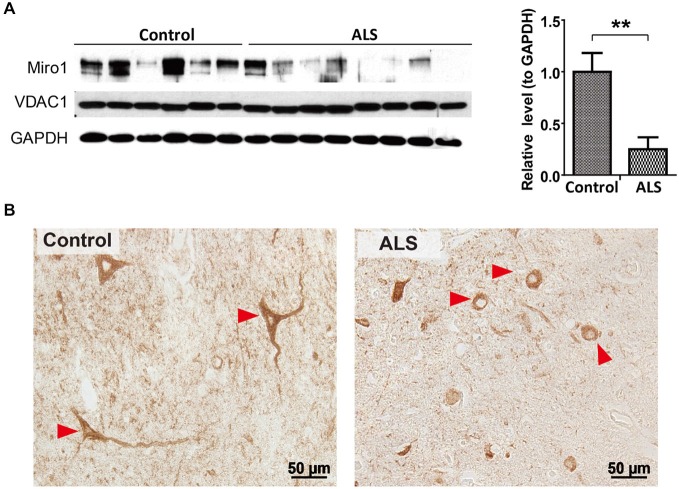
**Expression of Miro1 in spinal cords of ALS patients. (A)** Representative immunoblot and quantification analysis of Miro1 expression in thoracic spinal cord tissues from sporadic ALS (sALS) patients (*n* = 8) and age-matched control subjects (*n* = 6). Equal protein amounts (10 μg) were loaded and confirmed by GAPDH. VDAC1 was used as a mitochondrial specific marker. **(B)** Representative immunocytochemistry of mitochondrial marker COXI in lumbar spinal cords of sALS patients and age-matched control subjects. Arrowheads denote representative COX1 staining in motor neurons. Data are means ± sem. Statistics: student *t* test. **p < 0.01, compared with control subjects.

We first investigated the expression of Miro1 in spinal cord tissues from 8 ALS patients and 6 age-matched normal subjects. Immunoblot analysis revealed that Miro1 protein levels were dramatically reduced in ALS spinal cords compared to age-matched control spinal cords (Figure 1A). No significant changes in overall mitochondrial content were noted between ALS and control samples as evidenced by the constant expression levels of the mitochondrial marker VDAC1 (Figure 1A). We further performed immunocytochemical analysis of mitochondrial distribution in motor neurons using a specific antibody against mitochondrial marker, cytochrome c oxidase subunit 1 (COX-1). Consistent with the previous study showing impaired mitochondrial transport in ALS (Sasaki and Iwata, 1996), COX-1 immunoreactive mitochondria were only present in soma and depleted in the neurites of spinal cord motor neurons of ALS patients, compared to the uniform distribution of Cox-1 throughout the soma and neurites of age-matched control motor neurons (Figure 1B).

### Reduced Expression of Miro1 in Spinal Cords but not Brains of SOD1G93A Transgenic Mice

**Figure 2:**
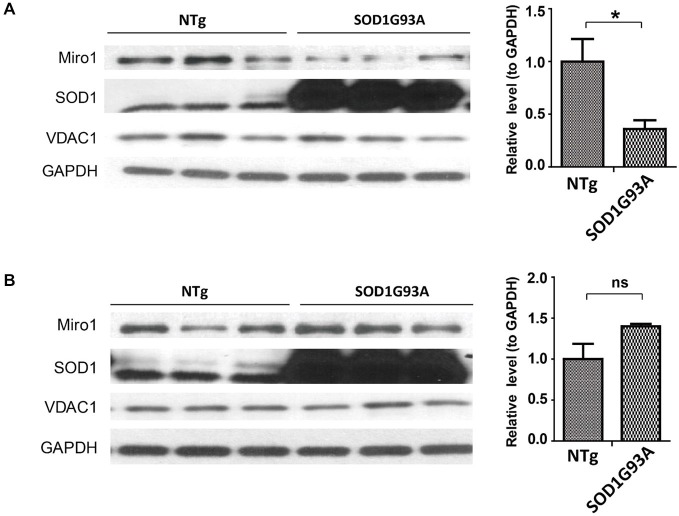
**Expression of Miro1 in spinal cords and brains of SOD1G93A mice**. Representative immunoblot and quantification analysis of Miro1 expression in spinal cord **(A)** and brain **(B)** of 60 day old SOD1 G93A mice and age-matched non-transgenic mice (NTG). *N* = 3 male mice/group. Equal protein amounts (10 μg) were loaded and confirmed by GAPDH. VDAC1 was used as a mitochondrial specific marker. Experiments were repeated three times. Data are means ± sem. Statistics: student *t* test. *p < 0.05, compared with control subjects. ns, non-significant.

Since the widely used transgenic mice expressing ALS-associated SOD1 G93A mutant (SOD1G93A mice) develop phenotypes closely mimicking fALS and sALS, we next studied the expression of Miro1 in the spinal cord and brain tissues from these mice. Previous studies showed that ALS disease progression went through different stages ending in paralysis in SOD1G93A mice, with phenotype only evident after 90 days of age (Kong and Xu, 1998). Compared with age matched control non transgenic mice (NTg mice), the expression of Miro-1 in spinal cords was decreased significantly even in 60 day old SOD1G93A mice, well preceding the onset of any disease stage (Figure 2A). Surprisingly, unlike what we observed in the spinal cord tissues, the Miro1 level in brains of SOD1G93A mice remained unchanged and was similar to NTg mice (Figure 2B).

### Reduced Expression of Miro1 in Spinal Cords but not Brains of TDP-43M337V Transgenic Mice

**Figure 3:**
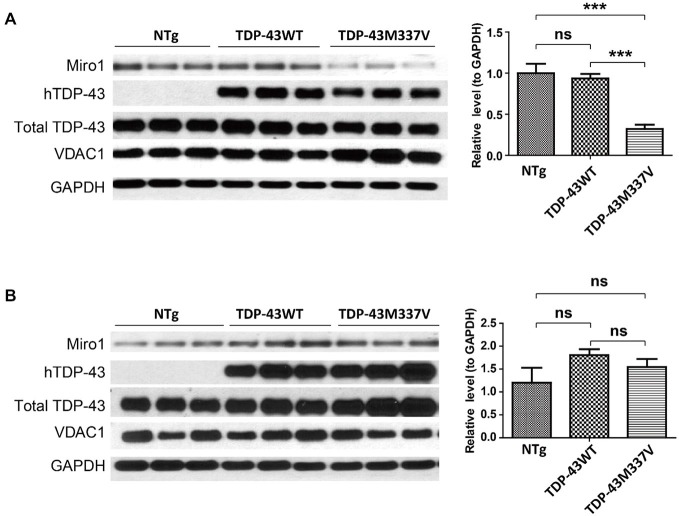
**Expression of Miro1 in spinal cords and brains of TDP-43M337V mice**. Representative immunoblot and quantification analysis of Miro1 expression in spinal cords **(A)** and brains **(B)** of 60 day old male TDP-43M337V mice and age-matched NTG mice or TDP-43WT mice. *N* = 3 male mice/group. Equal protein amounts (10 μg) were loaded and confirmed by GAPDH. VDAC1 was used as a mitochondrial specific marker. Experiments were repeated three times. Data are means ± sem. Statistics: one-way ANOVA followed by Tukey’s multiple comparison test. ***p < 0.05, compared with control subjects. ns, non-significant.

Autosomal dominant mutations in TDP-43 are associated with both sporadic and familial ALS (Kabashi et al., 2008; Sreedharan et al., 2008). We further sought to investigate the expression of Miro1 in transgenic mice expressing ALS-associated TDP-43 M337V mutant (TDP-43M337V mice). Interestingly, Miro1 was also found to be greatly reduced in spinal cords of TDP-43M337V mice when compared with age matched control NTg mice or transgenic mice expressing wild type TDP-43 (TDP-43WT mice) (Figure 3A). As we found in the SOD1G93A mice, brain samples of TDP-43M337V mice also demonstrated a similar expression level of Miro-1 to NTg or TDP-43WT mice (Figure 3B).

### Miro1 Deficiency in sALS was Recapitulated by Treating Motor Neurons with Glutamate

**Figure 4:**
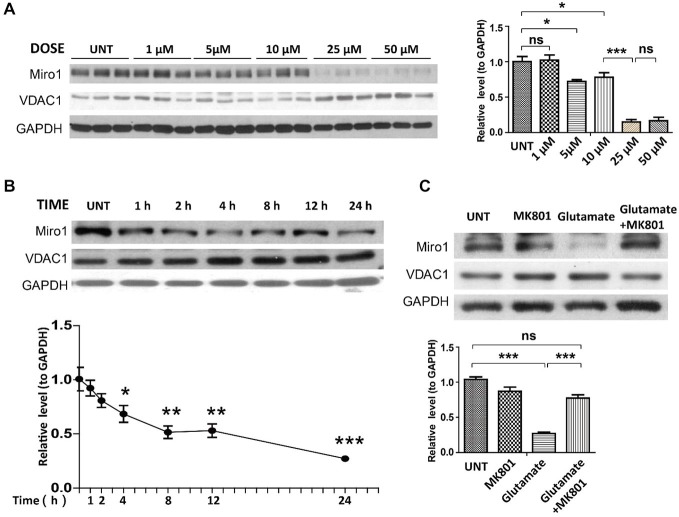
**Expression of Miro1 in spinal cords motor neurons treated with glutamate. (A)** Representative immunoblot and quantification analysis of the expression of Miro1 in motor neurons (DIV 5) treated with different doses of glutamate for 24 h. **(B)** Representative immunoblot and quantification analysis of the expression of Miro1 in motor neurons (DIV 5) at different time points after 25 μM glutamate treatment. **(C)** Representative immunoblot and quantification analysis of the expression of Miro1 in motor neurons treated with 25 μM glutamate and/or 20 μM MK801 for 24 h. Equal protein amounts (10 μg) were loaded and confirmed by GAPDH. Data are means ± s.e.m. All experiments were repeated three times. Statistics: one-way ANOVA followed by Tukey’s multiple comparison test. *p < 0.05, **p < 0.01, ***p < 0.001, compared with untreated control neurons.

Many ALS patients demonstrate elevated extracellular glutamate levels in the spinal cord, and glutamate excitotoxicity has been implicated in the pathogenesis of both sALS and fALS (Rothstein, 1995; Bruijn et al., 2004; Jaiswal, 2013). We further employed rat primary spinal cord cultured motor neurons challenged with glutamate as a model to investigate the potential cause of Miro1 deficiency in sALS. Primary motor neurons (day *in vitro* 5: DIV 5) were treated with different doses of glutamate (0–50 μM) for 24 h. Treatment with glutamate resulted in a significant dose-dependent decrease of Miro1 in spinal cord motor neurons starting at a concentration of 5 μM (Figure 4A). A further time course study using a sublethal dose of glutamate (25 μM) revealed that Miro1 levels were decreased within 2 h following glutamate treatment (Figure 4B), preceding neuronal death that was only observed 8 h later (Wang et al., 2015) (data not shown). As expected, and consistent with previous studies (Ackerley et al., 2000; Donnelly et al., 2013), this glutamate-induced Miro1 reduction was completely abolished by glutamate receptor blocker MK-801 (Figure 4C).

### Reduced Expression of Miro1 in Spinal Cords but not Brains of Mice with Intraventricular Glutamate Infusion

**Figure 5:**
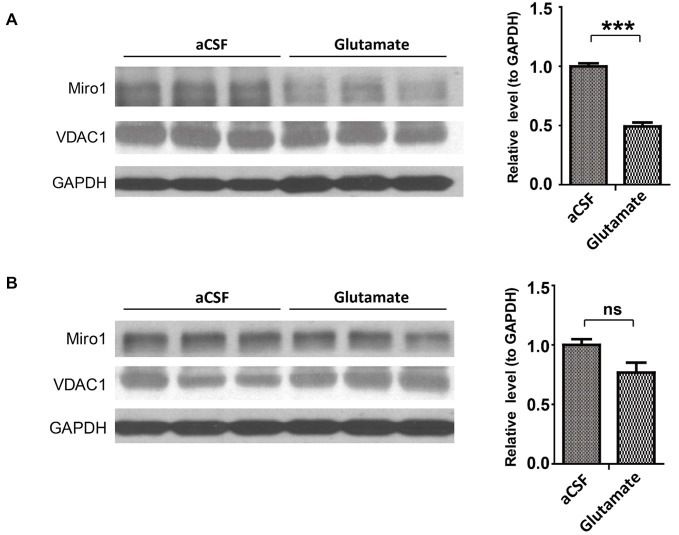
**Expression of Miro1 in spinal cords and brains of mice infused with glutamate**. aCSF containing 10 mM glutamate was continuously delivered into the lateral ventricle of 4–6 month old male non-transgenic wild type mice by mini-osmotic pumps (1 μl/h flow rate) for 7 days. Representative immunoblot and quantification analysis of Miro1 expression in spinal cords **(A)** and brains **(B)** of glutamate infused mice or age matched aCSF infused control mice. *N* = 3 mice/group. Equal protein amounts (10 μg) were loaded and confirmed by GAPDH. VDAC1 was used as a mitochondrial specific marker. Experiments were repeated three times. Data are means ± sem. Statistics: one-way ANOVA followed by Tukey’s multiple comparison test. ***p < 0.001, compared with control subjects. ns, non-significant.

As glutamate caused Miro1 reduction in *in vitro* cultured spinal cord motor neurons, we further attempted to confirm this findings in spinal cord motor neurons *in vivo*. NTg mice received continuous administration of a low dose of glutamate (10 mM at a flow rate of 1μl/h) into the left lateral ventricle via osmotic mini-pumps for 7 days. Continuous glutamate infusion did not result in death or any histological changes of brain, spinal cord and muscle (data not shown). Compared with artificial cerebrospinal fluid (aCSF) infused control mice, mice treated with glutamate demonstrated significant reduction of Miro-1 in spinal cords (Figure 5A). In addition, and again consistent with results observed in SOD1G93A and TDP-43M337V mice, the expression of Miro1 in brain tissue was unaltered by glutamate infusion (Figure 5B).

## Discussion

Here we show that the expression of Miro1 is significantly reduced in the spinal cords of sALS patients, SOD1G93A mice, TDP-43M337V mice and mice infused with glutamate. An unexpected finding of this study is that Miro-1 remains unchanged in the brains of all animal models, which may help to explain the selective vulnerability of motor neurons in the spinal cord. On the mechanistic level, we demonstrated that glutamate exposure leads to dose and time dependent decrease of Miro1 in cultured spinal cord motor neurons, which was blocked by NMDA receptor antagonist.

The key finding of this study is the consistent motor neuron specific Miro1 deficiency in human disease (sALS patients), and mouse models of ALS (SOD1G93A mice and TDP-43M337V mice). Previous studies reported that mitochondrial movement and distribution were impaired in ALS experimental models (De Vos et al., 2007; Bilsland et al., 2010; Wang et al., 2013; Magrané et al., 2014). Because Miro1 is critical for kinesin-mediated mitochondrial movement, these findings suggest that Miro-1 deficiency may be responsible for mitochondrial movement deficits in ALS and ALS experimental models. Since the expression of Miro-1 in brain is not altered by expression of SOD1 G93A or TDP-43 M337V in mice, Miro-1 deficiency is likely specific to motor neurons. Given the additional fact that Miro1 is reduced in young SODG93A mice well preceding disease onset and the fact that mice with Miro1 deficiency show motor deficits (Nguyen et al., 2014), all these studies together suggest that Miro-1-mediated mitochondrial movement deficits might be the cause rather than the consequence of ALS. It is still unclear why the expression of Miro-1 in brains remained at a steady state in SOD1G93A and TDP-43M337V mice and it would be interesting to investigate the role, if any, Miro1 plays in mitochondrial movement or distribution in brain neurons in these mice. Nevertheless, further studies to test whether overexpression of Miro1 can improve mitochondrial/neuronal function and phenotypes of either SOD1G93A or TDP-43M337V mice will provide insights into the potential role of Miro1-mediated mitochondrial abnormalities in the onset and progression of ALS.

Miro1 deficiency is noted in sALS patients and mice expressing fALS-associated SOD1G93A or TDP-43M337V, suggesting sALS and fALS may share common molecular processes through Miro1 deficiency. One such candidate process may be either elevated glutamate or problems in both glutamate-receptor and glutamate-transporter systems that have been repeatedly reported in many cases of sALS and fALS (Rothstein, 1995; Bruijn et al., 2004; Jaiswal, 2013). Indeed, in this study we found Miro1 clearly decreased in cultured spinal cord motor neurons treated with glutamate. Consistently, *in vivo* glutamate-induced Miro1 deficiency also occurs in spinal cords, but not brains, of mice infused with glutamate. These results provide direct support for the hypothesis that glutamate excitotoxicity might be the cause of Miro1 deficiency in sALS. Some previous studies revealed that either glutamate transporters or glutamate-transport activities were reduced in transgenic mice expressing ALS-associated SOD1 mutants, indicating the involvement of glutamate excitotoxicity in mediating ALS-mutant SOD1 neuronal toxicity (Bruijn et al., 1997; Bendotti et al., 2001; Warita et al., 2002; Boston-Howes et al., 2006; Yamanaka et al., 2008). Therefore, glutamate excitotoxicity might be a common mechanism leading to Miro1 deficiency in both sALS and fALS. SOD1 is present on mitochondria (Kawamata and Manfredi, 2010). It is also possible that SOD1 might directly interact with mitochondrial movement machinery to regulate mitochondrial movement and distribution.

Increasing evidence suggest a critical role of mitochondria in the pathogenesis of ALS and other neurodegenerative diseases including Alzheimer’s disease, Parkinson’s disease, Huntington’s disease (Shi et al., 2010; Su et al., 2010; Archer, 2013). The proper intracellular distribution of mitochondria adapting to localized bioenergetic requirement is critical for cellular physiology (Frazier et al., 2006). As Miro1 is essential for the regulation of mitochondrial mobility, the Miro1 deficiency observed in this study in *in vivo* and *in vitro* models of ALS and in human disease tissue, is likely a major mediator for impaired mitochondrial movement and distribution in ALS. The suppression of Miro1 reduction might be a potential novel therapeutic approach worthy of continued investigation and validation in ALS experimental models.

## Conflict of Interest Statement

The authors declare that the research was conducted in the absence of any commercial or financial relationships that could be construed as a potential conflict of interest.
